# Strategic Patenting by Pharmaceutical Companies – Should Competition Law Intervene?

**DOI:** 10.1007/s40319-020-00985-0

**Published:** 2020-10-28

**Authors:** Olga Gurgula

**Affiliations:** grid.7728.a 0000 0001 0724 6933 PhD; LLM; Lecturer in Intellectual Property Law at Brunel Law School, Brunel University London, Uxbridge, UK

**Keywords:** Strategic patenting, Evergreening, Pharmaceutical patents, Anticompetitive effect, Generic competition, Access to medicines, Access to COVID-19 treatment

## Abstract

As the COVID-19 pandemic is affecting the lives of thousands of people worldwide, the problem of timely access to affordable medicines has intensified today. Based on past experience of accessing medicines for life-threatening diseases there is a justifiable fear that access to any vaccines and treatments that are eventually developed may be hindered by patents, leading to unaffordable prices. In particular, one of the reasons that typically leads to high prices is strategic patenting employed by pharmaceutical companies. While this practice is currently considered lawful, this article argues that strategic patenting requires a long-overdue intervention by competition authorities and aims to attract their attention to its harmful effects. It maintains that, along with a more immediate negative effect in the form of high drug prices, strategic patenting affects dynamic competition by stifling innovation of both originators and generic companies. The article outlines the current approach to strategic patenting and provides arguments for the intervention of competition law. This, in turn, will open the possibility for competition authorities to investigate this practice and prevent its harmful effect on drug prices and pharmaceutical innovation, for the benefit of consumer welfare.

## Introduction

As the COVID-19 pandemic is sweeping through the world, thousands of people urgently need access to affordable medicines. Based on past experience of treatments for other life-threatening diseases, there is a fear that access to any vaccines and treatment that may be developed in the future will be affected by patents,^1^ leading to unaffordably high prices.^2^ However, the problem of high drug prices is not new. It had been inflating healthcare budgets and posing a serious risk to the affordability and accessibility of medicines for society well before the pandemic.^3^ This problem is further exacerbated by the fact that, despite the alleged surge in investments into pharmaceutical R&D, current statistics indicate that the number of new breakthrough medicines is decreasing.^4^ On the other hand, the number of drugs that contain modifications of existing medicines is growing, demonstrating that pharmaceutical companies have been increasingly focusing their research on incremental drug development, rather than on breakthrough innovation.^5^ Various reasons for high drug prices and the growing focus on incremental innovation are put forward by pharmaceutical companies, including the complexity of drug discovery and development, as well as the expensive and lengthy regulatory procedures involved.^6^ While these reasons play an important role in this regard, some practices by pharmaceutical companies substantially contribute to this problem.^7^ In particular, pharmaceutical companies have been increasingly engaging in strategic patenting to delay or even block generic competition.^8^ These practices attracted the attention of the European Commission, which discussed them more than a decade ago in its 2009 Pharmaceutical Sector Inquiry Report.^9^ The Commission identified a series of patent strategies which it described as aiming “to extend the breadth and duration of [originators’] patent protection”^10^ and “to delay or block the market entry of generic medicine”.^11^ Such findings have fuelled debates as to whether these strategies may be deemed unlawful and violate EU competition rules, while also being justifiable business practices under patent law. Until today, no agreement has been reached either on the legality of these practices, or on an efficient legal tool to assess them. As a result, despite there being solid evidence that such strategies may block generic competition, allowing originators to maintain artificially high drug prices and preventing patients from accessing cheaper generics, they remain outside the ambit of the Commission’s activities. Instead, the Commission has been focusing on more straightforward patent-related practices, such as reverse payment agreements.

This article argues that strategic patenting by pharmaceutical companies requires a long-overdue intervention by competition authorities. It aims to attract their attention to the harmful effects of strategic patenting. Specifically, it will contest the argument traditionally put forward by originator pharmaceutical companies that the intervention of competition law into patenting practices will reduce their incentives to innovate. The paper will argue to the contrary that, along with a more immediate negative effect in the form of high drug prices that is widely explored in the literature,^12^ strategic patenting also affects dynamic competition by stifling innovation. Importantly, it will be explained that the assessment of the effect of this practice should focus not only on innovation by originators, but should also take a wider market perspective by assessing its effect on follow-on innovation by generic companies. The latter argument is often overlooked. The paper will outline the current approach to strategic patenting that considers this practice lawful, and will provide arguments for the intervention of competition law. This, in turn, will open the possibility for competition authorities to investigate this practice in order to prevent its harmful effect on innovation and consumer welfare. Moreover, while patent law may provide certain mechanisms to deal with strategic patenting, such as raising the bar for patentability of pharmaceutical follow-on inventions,^13^ these tools may not be effective in all cases. Therefore, as will be explained further, competition law may be a more suitable tool to address the negative effects of strategic patenting.^14^

The article will be organised as follows. It will first discuss the complex structure of the pharmaceutical industry, focusing on its key players for the purpose of this article: originators and generic companies. It will further explore patenting practices employed by pharmaceutical companies and will define the notion of strategic patenting. The article will then argue that the latter strategy is against the rationale of patent and competition laws, as it stifles competition by impairing incentives to innovate of both originators and generic companies. Finally, it will discuss the current approach to strategic patenting that considers this practice lawful, and will argue that it should be subject to scrutiny under the rules of competition law, to address its negative effects.

## Pharmaceutical Innovation and Generic Competition in the Pharmaceutical Industry

The pharmaceutical industry is unique in its complexity. It is characterised by heavy state regulation and, sometimes, by the competing interests of the pharmaceutical business and society. It also involves multiple actors, including originators,^15^ marketing authorisation bodies, generic companies,^16^ doctors, pharmacies and patients. Each of them plays their part in the lengthy and complicated process of transforming a chemical compound into an effective and affordable medicine, which is then prescribed, dispensed and consumed. In these complex relationships, the two key players have crucial roles. On the one hand, originators play an important role in developing new and improved medicines for the benefit of society. On the other hand, generic companies benefit society by supplying cheaper equivalents of the originators’ medicines, which leads to the reduction of drug prices and facilitates access to affordable medicines. When the interests of these two players are kept in balance, benefits are maximised for society, which receives innovative and improved medicines, as well as timely access to generic drugs. However, if the balance swings towards one of the players, then society loses out, as there will be insufficient access to either innovative or affordable medicines. Therefore, both pharmaceutical innovation and generic competition must be duly incentivised and protected.

Moreover, these two elements of the pharmaceutical industry are constantly interacting and have a profound impact on each other. In particular, pharmaceutical innovation is the backbone of the pharmaceutical industry, in which originators play an important role. The process of drug development is long and complicated, requires significant investments, and bears considerable commercial risks.^17^ It is also highly regulated, including, among other things, the requirement for originators to obtain a special authorisation from a designated state authority to market a drug. Such marketing authorisations are granted to the originators only if they can prove that the drug is safe and effective, which typically requires lengthy and expensive clinical trials.^18^

In order to protect these significant efforts and investments, pharmaceutical companies rely heavily on the exclusivity granted by intellectual property rights, and in particular, patents.^19^ Patents provide a 20-year monopoly right, during which a pharmaceutical company enjoys market exclusivity and can charge a monopoly price for its products. Originators argue that strong patent protection is essential in order to recoup investments, as well as to incentivise them to engage in further innovation.^20^ Once such patent protection expires, however, other companies may develop generics of a branded drug, and start competing with the originator for the market. This is called generic competition. Generic drugs are bioequivalent versions of a branded drug that has lost its patent protection.^21^ It is estimated that the generic entry typically leads to, on average, an 80 per cent market share loss and a 20–30 per cent reduction of a drug price, with further price decreases with each additional generic entrant, leading, in some instances, to a fall in price of up to 90 per cent.^22^ A representative example of the effect of generic competition on the originators’ drug prices is the significant decrease in price and dramatic loss of profits by Eli Lilly. The expiration of a patent protecting its blockbuster^23^ antidepressant Prozac in 2001 resulted in a loss of almost 70 per cent of its market and $2.4 billion in annual U.S. sales.^24^ This effect of generic competition is beneficial for society, as it reduces the financial pressure on healthcare budgets and increases the accessibility of drugs.

## Patenting Practices by Pharmaceutical Companies

As was mentioned above, generic competition is prevented during the life of a patent protecting an active compound of a drug (a so-called “basic” or “primary” patent).^25^ Such a basic patent covers an active ingredient itself and, therefore, provides the strongest protection for the product. Therefore, generic competition normally starts only after the basic patent expires, or if a generic company succeeds in invalidating it. While in the past pharmaceutical companies mainly protected their products with a single patent covering an active compound,^26^ they now increasingly seek additional patent protection on various aspects of a drug^27^ in order to protect their market position.^28^ Such additional patents are often called secondary patents.^29^ A pharmaceutical company may want to obtain secondary patents, which protect such aspects of a drug as, for example, its process of manufacture, formulation and/or specific form, etc. Therefore, even after the basic patent protecting an active compound expires, a drug may still be protected by other secondary patents. This may result in the extension of the scope and length of the protection of a product, especially if secondary patents have a later expiration date than a basic patent.^30^ This, in particular, may occur if, for example, the process of producing an active compound disclosed in the basic patent is sufficient only for reproducing this compound in a laboratory, but it is unsuitable for producing it on a large commercial scale.^31^ If the originator was able to secure a secondary patent that protects such a large scale manufacturing process, it would prevent generics from using this process for producing their generic versions of a drug; otherwise they would risk infringing this secondary patent.^32^ However, a unique feature of pharmaceuticals is that an active ingredient can be manufactured using different methods and processes, can exist in different forms or can be used in different formulations. Therefore, when a basic patent on an active ingredient expires, other companies can develop alternative methods of production, forms or formulations of this active compound and start competing with the originator company.^33^ While such patenting strategies by originators are lawful in principle, some of them may be problematic. In particular, in anticipation of the loss of patent protection, originators may engage in strategic patenting which artificially prevents generic competition and results in an extension of their market monopoly.^34^

## Defining Strategic Patenting

In its Sector Inquiry Report, the European Commission explained that the drug development process consists of three main stages: (i) the R&D stage, which ends with the launch of a drug on the market; (ii) the period between the launch and the patent expiry; and (iii) the period after the patent expiration, when generics can enter the market.^35^ During the second stage, i.e. after the launch of a drug, originators seek to maximise their income from the product in order to recoup their R&D investments and earn profits before the commencement of generic competition.^36^ It is also during this stage that pharmaceutical companies seek to prolong their market exclusivity.

In recent years, pharmaceutical companies have been increasingly relying on the strategic use of the patent system to combat the pressure of generic competition. Such practices are often called “life cycle management” by originators and proponents of the practice. For example, as Burdon and Sloper explained, “[a] key element of any life cycle management strategy … is to extend patent protection beyond the basic patent term for as long as possible, by filing secondary patents which are effective to keep generics off the market”.^37^ However, critics have characterised the practice as “evergreening”,^38^ as it essentially evergreens the patent protection and the exclusivity of a product.^39^ For instance, Bansal et al. explain that evergreening “refers to different ways wherein patent owners take undue advantage of the law and associated regulatory processes to extend their IP monopoly, particularly over highly lucrative ‘blockbuster’ drugs, by filing disguised/artful patents on an already patent-protected invention shortly before expiry of the ‘parent’ patent”.^40^

During its investigation into the pharmaceutical industry, the European Commission found that the number of patents granted and pending applications significantly increases with the value of a drug, i.e. “blockbuster medicines can even be protected by up to nearly 100 INN^41^-specific EPO patented bundles and applications …, which in one particular case led to 1,300 patents and applications across all the EU Member States”.^42^ The Commission also found that the ratio of primary to secondary patents is 1:7, where the latter “mostly concern formulations, processes and non-formulation products…, such as salts, polymorphic forms, particles, solvates and hydrates”.^43^ As a result, the Commission concluded that the practice of “maximising patent coverage in such a way is the creation of a web of patents”, which affects the generics’ ability to “develop a generic version of the medicine in form of a salt, crystalline or amorphous form”, because it “would inevitably infringe a patent (for example, a patent for the relevant salt, crystalline or amorphous form of the medicine)”.^44^ Each of such patents would typically have a later expiration date, which effectively extends a period of market exclusivity beyond the expiration of a basic patent.^45^ In addition, most of these patents that protect such follow-on modifications are so-called “sleeping” patents, i.e. patents which a company has no intention of commercialising.^46^ Moreover, such modifications may provide little or no therapeutic benefits to the patient compared to the original drug.^47^ Nevertheless, such patents allow originators to secure the most efficient, broadest and longest possible protection for their successful products.^48^

The denser the web of secondary patents, the more difficult it is for generics to develop their generic equivalents, even if they know that only a few patents of a large portfolio would, in fact, be valid and infringed by their products.^49^ Despite such knowledge, it is impossible to be certain before introducing a generic whether this will be the case and, thus, whether the generic company will be subject to injunctions preventing the sale of their generic products.^50^ Such practice, therefore, provides an appreciable competitive advantage for originators by creating a significant legal and commercial uncertainty for generics in relation to the possibility of their market entry.^51^

This paper argues that such a strategic use of the patent system by pharmaceutical companies is against the shared goal of patent and competition laws of facilitating innovation for the benefit of society. As will be explained further, in addition to a more immediate negative effect in the form of high drug prices, strategic patenting may also impair innovation by reducing originators’ incentives to innovate, and affecting generics’ ability to develop alternative generic products. Strategic patenting, therefore, may enable originators to avoid competitive pressures by preventing generic competition without a need to engage in genuine innovation.

## Strategic Patenting Contradicts the Rationale of the Patent System and Competition Law

In the competitive markets, the success of a company is based on its business performance.^52^ In order to compete on performance by “offering better quality and a wider choice of new and improved goods and services”^53^ firms must innovate. Realising the importance of protecting innovation, which is considered to be the main driver of economic growth,^54^ states have put in place various mechanisms to ensure a suitable environment for its advancement. These include granting the property rights to the results of innovation in the form of patents, as well as implementing competition law rules to stimulate dynamic competition.^55^

Specifically, one of the main justifications for the patent system is the encouragement of innovation^56^ that serves as an engine for economic growth and development.^57^ The patent system pursues this aim by offering the patent owners a period of exclusive rights as a reward for their innovative efforts and an incentive to engage in further innovation.^58^ Therefore, intellectual property rules, and patents in particular, are seen as an essential element of undistorted competition on the internal market.^59^ These exclusive rights are considered to be a necessary incentive to invest in R&D and innovation, particularly in such sectors as pharmaceuticals, where the R&D costs are high, but the costs of copying the R&D results are marginal.^60^ At the same time, the “innovation theory”, embodied in the EU competition law rules and policy, is designed to stimulate innovation by fostering competition on the markets.^61^ The competition law rules keep markets innovative by maintaining effective competition through preventing the foreclosure of markets and maintaining access to them.^62^ The rationale is that firms react to pressures of competition by continuously seeking to innovate.^63^ Therefore, patent and competition laws complement each other, as on the one hand, existing competition creates pressures on firms, forcing them to innovate, the so-called “stick”, while on the other hand, patent law provides a “carrot” in the form of the exclusive right, thus inducing innovators to innovate.^64^ These two bodies of laws are seen as “complementary efforts to promote an efficient marketplace and long-run, dynamic competition through innovation”.^65^ As the European Commission noted “both intellectual property rights and competition are necessary to promote innovation and ensure a competitive exploitation thereof”.^66^ These two bodies of laws, therefore, have the same fundamental goal of enhancing innovation for the benefit of consumer welfare.

Importantly, patent and competition laws are designed to stimulate not only innovation of “pioneer” innovators, but they are also aimed at facilitating follow-on innovation.^67^ Patent law contains provisions that require inventors to disclose information about their inventions, as well as providing exceptions such as experimental use and compulsory licensing, which allow third parties to access the inventions still under patent protection.^68^ Therefore, along with pioneer innovators, the rationale of incentives to innovate in patent law also applies to follow-on innovators, balancing the interests of these two types of inventors.^69^ Similarly, competition law aims at stimulating all types of innovation, including follow-on innovation.

On the other hand, EU competition law proscribes practices that reduce incentives to innovate both for “pioneer” and follow-on innovators. This is enshrined in Art. 102(b) TFEU, which prohibits abuses that consist of, *inter alia*, limiting technological development. For example, in *AstraZeneca* the General Court considered that the company’s practice of misusing the patent system had the potential of reducing its incentives to innovate and was anticompetitive.^70^ In *Magill*^71^ and *Microsoft*,^72^ the courts found that the IP rights owners abused their dominant positions by blocking innovation of their potential competitors. More recently, several decisions by the European Commission also emphasised the importance of protecting innovation. In January 2018, the Commission fined Qualcomm^73^ €997 million for abusing its market dominance in LTE^74^ baseband chipsets.^75^ The Commission considered that the exclusivity payments that Qualcomm paid to Apple denied rivals the possibility to compete on the merits, and deprived European consumers of genuine choice and innovation.^76^ Furthermore, in July 2018, the Commission found in *Google Android* that Google abused its dominant position, and fined the company €4.34 billion for anticompetitive restrictions it had imposed on mobile device manufacturers and network operators to strengthen its dominant position in general internet search.^77^ The Commission considered that Google’s restrictive practices denied other companies the chance to compete on the merits and innovate.^78^ Finally, in 2017 the Commission issued its decision, in which it took the view that Amazon abused its dominant positions on the markets for the retail distribution of e-books by inserting the so-called “parity clauses” in the agreements with its e-book suppliers.^79^ It concluded that these clauses had the potential of reducing the incentives to innovate both by e-book suppliers and retailers.^80^

These decisions demonstrate that the European Commission recognises the fundamental importance of protecting innovation. They confirm that strategies that are capable of stifling innovation and reducing the incentives to innovate may constitute an abuse of dominance under Art. 102 TFEU. It is argued in this article that, along with the practices condemned by the Commission in the decisions discussed above, strategic patenting can also harm innovation by impairing incentives to innovate of both originators and generic companies, and therefore should raise competition law concerns.

### Strategic Patenting Impairs Originators’ Incentives to Innovate

While originator companies typically argue that the competition law intervention into their patenting practices will reduce their incentives to innovate,^81^ this article asserts that strategic patenting itself reduces originators’ incentives. Thus, in a properly functioning system, when a patent protecting a product is close to expiration the originator would be encouraged to innovate further in order to introduce a new product on the market and maintain its competitive position. However, by engaging in strategic patenting, the originator’s incentive to innovate diminishes as it enjoys its monopoly position by merely procuring numerous secondary patents that shield its current product from generic competition. Therefore, when companies engage in such strategic patenting, they are merely protecting themselves from the competitive pressures that competition law aims to establish.

Maintaining that this practice is lawful, originators argue that strong patent protection is essential for recouping their investments, as well as for incentivising them to engage in further innovation.^82^ Such a position may find some support in the arguments put forward by Joseph Schumpeter and his followers, who claimed that since monopoly increases the reward of the innovator, monopolists are more prone to innovation.^83^ However, as Lowe noted:^84^

the empirical evidence of the past few decades has worked against Schumpeter and in favor of Kenneth Arrow, who contends that in favoring monopolies Schumpeter underestimated the incentives for innovation that competition can offer. Monopolists tend to want to keep their monopolies by resorting to any measures that can keep new entrants out. Firms under competitive pressure from actual or potential competition, on the other hand, are less complacent and know that inventing a new product is their best strategy for maintaining and increasing their market share.

In the same vein, the Commission emphasises the importance of competition for the incentives to innovate, stating that: “[r]ivalry between undertakings is an essential driver of economic efficiency, including dynamic efficiencies in the form of innovation. In its absence the dominant undertaking will lack adequate incentives to continue to create and pass on efficiency gains.”^85^

Evidence from the pharmaceutical industry confirms that strategic patenting reduces incentives to engage in genuine and meritorious innovation. In many cases, strategically accumulated secondary patents are of marginal quality and are typically the result of routine research activities.^86^ For example, in *Perindopril* the European Commission revealed that most of the secondary patents, procured as part of the originator company’s anti-generic strategy, were seen by the company as “blocking” or “paper”, some of which it considered involved “zero inventive step”^87^ and a purely editorial task.^88^ Moreover, these follow-on pharmaceutical inventions are specifically timed around the expiration of the basic patent and can be developed on demand.^89^ In *AstraZeneca* the Commission noted that the company designed to “[f]ile a patent-cloud of mixtures, uses, formulations, new indications, and chemistry” in relation to its blockbuster product omeprazole to slow down generic entry at a specifically defined time, close to the expiration of the basic patent.^90^ The main aim of these patents is to increase uncertainty for generic companies as to the possibility of their market entry.^91^ Therefore, while many of these secondary patents may be trivial and potentially invalid, the originator pursues them to protect its current successful product from generic competition.^92^

Even if a company continues to engage in innovation in parallel to pursuing strategic patenting, it still protects itself from the pressures of competition, which would have forced the company to innovate faster and would thus provide consumers with better products and/or access to cheaper generic versions earlier. As Ullrich argues:^93^

A slowdown in the transition of the new medicines from the protected status of a proprietary medicine to the status of generic products manufactured and distributed in open competition does not simply mean a loss of static efficiency, namely a loss of consumer well-being due to a slowdown in the reduction of process. Rather, such a slowdown also involves the risk of a loss of dynamic efficiency in that it extends the duration of a monopoly rent situation, thus reducing the pressure to innovate more quickly.

Following the rationale of the General Court’s statement in *AstraZeneca*, the practice of the originator that extends its market monopoly by relying on the patent system “potentially reduces the incentive to engage in innovation, since it enables the company in a dominant position to maintain its exclusivity beyond the period envisaged by the legislator”.^94^ Such practices, according to the Court, act “contrary to the public interest”.^95^ Therefore, the practice of strategic patenting that protects originators’ monopolies from competitive pressures and significantly reduces their incentives to engage in genuine innovation is contrary to the rationale of the patent system, has a significant negative effect on competition and should raise competition law concerns.

### Strategic Patenting Impairs Follow-on Innovation of Generic Companies

Strategic patenting also has a chilling effect on follow-on innovation by generic competitors in the form of developing alternative versions of an off-patent compound. As was discussed earlier, the expiry of a basic patent that protects an active compound facilitates generic competition. This is because even if the product is still protected by process, specific form or formulation patents, generic companies may develop alternative ways of producing or formulating the product and start competing with the originator. In the absence of strategically accumulated patents by the originator, generic companies are typically open to innovating to launch alternative generic products as soon as the basic patent expires. However, by pursuing strategic patenting, originators may discourage generics from engaging in follow-on innovation because of the uncertainty about the patent protection and a fear of infringing on one of the numerous patents.^96^ In its Sector Inquiry Report, the Commission cited the following quote from one of the originators:

The entire point of the patenting strategy adopted by many originators is to remove legal certainty. The strategy is to file as many patents as possible on all areas of the drug and create a “minefield” for the generics to navigate. All generics know that very few patents in that larger group will be valid and infringed by the product they propose to make, but it is impossible to be certain prior to launch that your product will not infringe and you will not be the subject of an interim injunction.^97^

Therefore, as a result of creating an impenetrable ring of patent protection by the originator,^98^ generic competitors may be prevented from developing alternative generic versions of an off-patent compound. One of the examples revealed by the Commission during its Pharmaceutical Sector Inquiry was the filing by an originator company of “more than 30 patent families translating into several hundreds of patents in the Member States in relation to one product”, many of which were filed after the introduction of the product.^99^ This affected the intentions of several generic companies that planned to develop and bring their generic versions of the original product to the market.^100^

As a result, in addition to the already high barriers to entry into the pharmaceutical market due to patents that protect an existing product and the need to obtain a marketing authorisation, strategic patenting raises these entry barriers further, making it very difficult for generic companies to overcome them. This strategy, therefore, “may without further enforcement action by originator companies, … delay generic entry until the patent situation is clearer or even discourage more risk-sensitive generic companies from entering altogether”.^101^ Consequently, the fact that actual or potential competitors of originators would not be able to develop alternative generic products means that no one could enter the market and challenge originators’ monopoly positions. This results in a weakening of competition in the relevant market and a strengthening of the originator’s already dominant position. As Maggiolino put it, “patent accumulation … may work as a pre-emptive entry-deterrence strategy to protect monopoly power and … lower consumer welfare by allowing dominant firms to keep on charging over-competitive prices”.^102^ Therefore, when an array of accumulated secondary patents “blocks monopolists’ rivals from producing follow-on innovations, this strategy prevents the whole society from enjoying … these further innovations”.^103^ While practices that facilitate innovation are encouraged by competition law, practices that are aimed at blocking follow-on innovation by competitors should raise competition law concerns.

## Strategic Patenting is Considered Lawful Under the Current Approach

As could be seen from the above discussion, strategic patenting acts against the rationale of the patent system and competition law as it impairs innovation, enabling pharmaceutical companies to block generic competition, which leads to high drug prices. However, in the EU, the courts have never applied the competition law rules to condemn the procurement of internally developed patents.^104^ This approach in the EU takes its origins from the “existence-exercise” doctrine.^105^ As the General Court explained, while the EU Treaties “never expressly provided for a reconciliation between intellectual property rights and competition law”, they

nevertheless provided for a reconciliation of intellectual property rights with the principle of free movement of goods, by indicating that the provisions of [Article 36 TFEU] relating to the prohibition of quantitative restrictions between Member States were not to preclude restrictions on imports, exports or goods in transit justified, *inter alia*, on grounds of the protection of industrial and commercial property.^106^

As a result, it is considered that this provision

intended to draw a distinction between the existence of a right conferred by the legislation of a Member State in regard to the protection of artistic and intellectual property, which cannot be affected by the provisions of the Treaty, and the exercise of such right, which might constitute a disguised restriction on trade between Member States.^107^

Therefore, as early as in 1974 it has been recognised that, while the conditions under which intellectual property rights are exercised may fall within the prohibitions contained in Arts. 101 and 102 TFEU, the existence of such rights is not affected by EU competition law.^108^

Following this reasoning, the EU competition case law does not deal with practices related to the existence of IP rights, and instead focuses on their exercise.^109^ Specifically, it provides that in order to establish an abuse of dominant position, a mere procurement of patents will not suffice, even if they have a negative effect on the market.^110^ To prove an abuse, an additional element in the form of unlawful exercise of IP rights, such as misuse of patent procedures,^111^ vexatious litigation^112^ or unlawful refusal to license,^113^ must be demonstrated. Recent case law confirms this approach. In *Perindopril*,^114^ the Commission investigated an anti-generic strategy of a pharmaceutical company, one of the crucial elements of which was the accumulation of blocking patents with the sole purpose of delaying generic competition. While having established that this practice had, in fact, such an effect, the Commission did not pursue it and instead focused on two other practices, namely the acquisition of alternative patents from third parties and reverse payment agreements. The Commission noted that a dominant firm “can have a strategy to protect its commercial interests without infringing Article 102 of the Treaty, which may particularly include the strategic use of IPRs and the patent system”.^115^ As a result, in its decision the Commission clearly distinguished patent strategies, i.e. strategic patenting in the form of accumulation of blocking patents from other practices, such as patent acquisitions and reverse payment agreements. The former strategies were seen as lawful, while the latter were considered anticompetitive.

Supporting the current approach to strategic patenting, some commentators argue that the internal mechanisms of patent law should deal with strategic patenting, and if this practice is in line with its rules and objectives, it must be considered lawful.^116^ This is justified by the idea that patent law facilitates innovation by granting inventors the right to exclude others from using their invention as a reward for their skills, creative efforts and investments.^117^ It is further argued that, from a patent law perspective, it is irrelevant whether the exclusion is based on one or thousands of patents, it is the value of innovation which they protect that matters.^118^ Therefore, it is maintained that strong patent protection promotes *ex ante* incentives to innovate and “patenting around the core invention increases the likelihood that protection will be effective and that the *ex ante* investment will be recouped”.^119^ As a result, it cannot be abusive to utilise the patent system to seek an optimal protection of an invention,^120^ and thus obtain multiple secondary patents around an active ingredient with the purpose of protecting it. In these circumstances, they explain, it is patent law and not competition law that must determine the question of a patent grant.^121^ Once patent law has determined that the invention merits protection, i.e. it meets the patentability criteria, competition law should not second-guess this decision.^122^

The proponents of the current approach further reject the Commission’s suggestion, put forward in its Sector Inquiry Report, that the intention to prevent generic competition may be important in determining whether the patenting practice is an abuse.^123^ It is argued that the intention of a dominant firm to exclude cannot be decisive, because the exclusionary effect is the very essence of a patent, and therefore “innovators that rely on patents to recoup their investments necessarily pursue a strategy of excluding entry”.^124^ It is considered that as far as competition law is concerned, the “right to exclude” allows the patent owner the right not to compete with himself.^125^ This would be the case even if it is done by removing competing patents from the market, accumulating them and keeping them dormant, rather than allowing others access to these patents.^126^ Therefore, since the ability to exclude is explicitly recognised in EU law as forming part of the specific subject matter of a patent, strategic patenting is “neither illegal nor anticompetitive”,^127^ and is immune from Art. 102 TFEU intrusion.^128^ In order to find an abuse, it is argued, there must be a “plus-factor”, such as, for example, fraud on the patent office.^129^ In line with this argument, other authors argue that this practice alone should not be of concern for competition law unless it is coupled with some additional anticompetitive practice, such as bundling and tying or mandatory package licensing.^130^

As a result, under the current approach the procurement of internally developed patents for the purpose of blocking generic competition is considered a lawful practice. However, as was discussed above, the patent system is used strategically to artificially block generic competition and prevent a timely arrival of cheaper generic versions. This, in turn, allows pharmaceutical companies to charge exorbitantly high prices on drugs, thus harming consumer welfare. Moreover, while some contend that the extension of the scope of Art. 102 TFEU application to patenting practices may have a significant negative effect on innovation,^131^ this article argued earlier that such practices, in fact, may stifle dynamic competition by impairing innovation, enabling dominant companies to strengthen their monopoly power.

## Strategic Patenting Should be Subject to Scrutiny Under Competition Law Rules

Considering the negative effect on drug prices and innovation, strategic patenting raises a crucial question: should it be shielded from any competition law interventions, or are there any instances where it may attract the attention of competition law? As noted above, the traditional approach to strategic patenting essentially prevents any competition law involvement. This position is based on several related arguments. It is considered that competition law should intervene only at the stage when IP rights are exercised, and should not concern itself with their mere existence. Furthermore, it is argued that strategic patenting is lawful because it is in line with patent law, and that internal patent law mechanisms should control this practice. As a result, in the absence of any additional factors, it should fall outside the oversight of competition law.^132^ This approach effectively prevents any competition law enforcement actions in relation to strategic patenting. However, as will be argued further, competition authorities may rely on the rules and principles of EU competition law to investigate this practice.

Thus, the first set of arguments against the competition law oversight of strategic patenting is firmly based on the “existence-exercise” dichotomy. However, while the traditional approach to strategic patenting considers that a mere procurement of patents cannot violate competition law, the support for a different view may be drawn from the *AstraZeneca* case. Specifically, *AstraZeneca* relates to the two abuses by the company that consisted of providing misleading information to the patent offices to obtain an extension of its patent rights in the form of supplementary protection certificates (SPCs) (first abuse); and the withdrawal of its marketing authorisation that prevented generics from relying on the data of the original medicine (second abuse). When assessing AstraZeneca’s conduct relevant to the first abuse, the courts dismissed the company’s argument that in order to find an abuse an IP right must be exercised, stating that the mere existence of such a right may also restrict competition.^133^ The General Court noted in this regard that:

[it] rejects the applicant’s argument that a finding of an abuse of a dominant position requires that an exclusive right obtained as a result of misleading representations has been enforced…. The mere possession by an undertaking of an exclusive right normally results in keeping competitors away, since public regulations require them to respect that exclusive right.^134^

The Court concluded that should it be otherwise it would tend to make the application of Art. 102 TFEU conditional on the infringements by competitors of a dominant firm’s IP rights.^135^ This finding is significant, since it essentially allows the Commission to overcome the “existence-exercise” doctrine, and, thus, may permit the enforcement activities in this practice.^136^

As to the second set of arguments against the involvement of competition law in strategic patenting, as discussed above, some authors argue that this practice is lawful because it is in line with patent law, and that the internal patent law mechanisms should control this practice, rather than the external rules of competition law. Such arguments, while sound, should not stop competition authorities from investigating strategic patenting. First, the current patent system in Europe allows and, in fact, encourages originators to procure secondary patents due to its low patentability standards. While many such patents may be sleeping patents, which bring no therapeutic benefit over existing medicines, they may be strategically used by pharmaceutical companies to prevent generic competition. Despite this, the patent system is not able to address this problem, as it does not control the intention of the patentee, contains no requirement to use the patent, and does not oblige the patentee to prove that its follow-on invention is better for the patient than the existing product (i.e. it merely requires that the invention is new and non-obvious, not superior). While one of the options could be to strengthen these patentability standards, which may reduce the number of secondary patents, this may not entirely solve the problem. This is because pharmaceutical companies may still be able to obtain patents on follow-on pharmaceutical inventions if they meet these patentability requirements. Moreover, it is unlikely that the jurisdictions with low patentability standards will raise the bar, because this would be against their policy objectives for setting the bar low in the first place in order to encourage pharmaceutical innovation. Finally, strengthening the inventive step requirement may potentially have an adverse effect, since in some cases pharmaceutical companies may choose to rely on trade secrets instead, which would result in many inventions remaining undisclosed. This article, therefore, submits that while the patent system is not adequately equipped to address the problem of strategic patenting, competition law may be a more suitable tool to deal with this practice.

Second, as to the lawfulness of this practice under patent law, it has long been recognised in EU competition law that “the illegality of abusive conduct under Article [102 TFEU] is unrelated to its compliance or non-compliance with other legal rules” and that “in the majority of cases, abuses of dominant positions consist of behaviour which is otherwise lawful under branches of law other than competition law”.^137^ Therefore, the fact that strategic patenting is lawful under patent law should not protect this practice from the competition law scrutiny.^138^ Furthermore, while it is true that patents provide the right to exclude others from using inventions, this, however, does not mean that the patent system may be used by a dominant firm as a tool for maintaining and strengthening its monopoly.^139^ As Ullrich argues, “the patent system is based on the idea of dynamic competition and not on the idea of competition to obtain a true monopoly covering the entire market, even if only temporarily”.^140^ In other words, once the procurement of patents by a dominant firm becomes a means for extending monopoly and harming competition, competition authorities must intervene.^141^

Based on these principles, developed in EU case law, this article argues that strategic patenting should be subject to scrutiny under the rules of competition law. Considering their significant negative effects, competition authorities should have the possibility to investigate not only practices related to the exercise of IP rights, but also strategies based on their existence, even if they are considered lawful under patent law. It is believed that the approach put forward in this article will allow the removal of artificial barriers to generic competition and, as a result, will facilitate access to affordable medicines.

## Conclusions

A patent gives its owner the legal right to exclude others from using his invention for a limited number of years, in exchange for disclosing the invention to the public. During the period of protection, the owner can recoup his investments in R&D; this acts as a reward for innovation, as well as an incentive to engage in further innovative activities. Patent protection is particularly important for the pharmaceutical industry. As the development of a drug is very expensive and time-consuming, pharmaceutical companies require sufficient protection to recoup their investments and earn profits, which in turn would allow financing further R&D. However, some pharmaceutical companies may use the patent system for a different purpose, i.e. they procure numerous secondary patents that create multi-layer protection around their successful products to avoid competition on the merits. Such strategic patenting allows pharmaceutical companies to strengthen their monopoly positions, and thus to continue charging high drug prices. Moreover, this article argues that such a practice is against the shared goal of patent and competition laws that are aimed at incentivising innovation, as it reduces the incentives for originators to innovate, harms follow-on innovation by generic companies and disturbs a fragile balance between patent monopoly and competition. It is, therefore, submitted in this article that while currently strategic patenting is considered a lawful practice, it should attract the attention of the European Commission and national competition authorities. Based on the principles developed in EU competition law, it is argued that strategic patenting should be subject to scrutiny under the rules of competition law. The approach put forward in this article will allow competition authorities to investigate strategic patenting, preventing its negative effect on competition and harm to consumer welfare.

## Footnotes

1 Prabhala and Hoen (2020) (“there is every indication that treatments for coronavirus may soon emerge, the mere fact of their existence is no guarantee that people will be able to access them. In fact, Covid-19 is more likely to end in the same way that every pandemic ends: treatments and vaccines will be buried in a thicket of patents – and pharmaceutical companies will ultimately make the decisions about who lives and who dies”. “Remdesivir, a medicine developed for Ebola by the biotechnology company Gilead, has major patents across the world that last until 2038”); Morten and Moss (2020); The Public Citizen (2020) (explaining that “Remdesivir, an experimental COVID-19 treatment, has benefited significantly from public funding”. Despite this the patents protecting Remdesivir are owned by Gilead).

2 Mancini (2020) (“Study argues prices for potential treatments are far higher than the actual cost of production”).

3 Kesselheim et al. (2016); UNCTAD (2015); Hubbard and Love (2004).

4 Pammolli et al. (2011); Scannell et al. (2012); Light and Lexchin (2012).

5 IMAK (2018), p. 3; Correa (2011), p. 2; Ho (2015), p. 312; Light and Lexchin (2012), p. 23 (“This is the real innovation crisis: pharmaceutical research and development turns out mostly minor variations on existing drugs, and most new drugs are not superior on clinical measures”).

6 EFPIA (2018), p. 6; Keyhani et al. (2005), p. 753; PHARMA (2015); DiMasi et al. (2003); *see*, however, Berman et al. (2017) (“Evidence has unequivocally shown that high drug prices are not linked to the actual costs of research, development and manufacturing. Instead, inflated drug prices are a result of drug manufacturers’ power to charge whatever price the market will bear”).

7 European Commission (2009a, b, c), pp. 181–379.

8 European Commission (2009a, b, c), pp. 181–202, 351–368.

9 European Commission (2009a, b, c).

10 European Commission, “Executive Summary of the Pharmaceutical Sector Inquiry Report” (2009a, b, c).

11 *Ibid*.

12 Kesselheim (2007); Kesselheim et al. (2016); Granstrand and Tietze (2015); IMAK (2018).

13 Gurgula (2019); other patent law mechanisms may include, *inter alia*, specific exceptions from patentability (Correa (2015), p. 18) and compulsory licensing (Art. 31 TRIPS).

14 This article focuses on the patenting practices related to small chemical compounds, rather than biological medicines. While strategic patenting may be employed in relation to both types of medicines, and hence the analysis in this paper may, to a certain extent, be relevant to biologics, specific patenting strategies may be different due to the nature of these technologies. In particular, while small chemicals can be classified as the discrete technology, biologics may be characterised as the complex technology. Hence, the approach to patenting, as well as to specific patent claims, may be different. Therefore, strategic patenting related to biologic drugs falls outside the scope of this article.

15 European Commission (2009a, b, c), p. 9 (“‘Originator company’ is defined as a company that sells originators, while an ‘originator’ is defined as a novel drug that was under patent protection when launched onto the market”).

16 European Commission (2009a, b, c), pp. 7–8 (“‘Generic company’ is defined as a company that sells generics, while a ‘generic’ is defined as a medicinal product which has the same qualitative and quantitative composition in active substances and the same pharmaceutical form as a reference (originator) medicinal product, and whose bioequivalence with the reference medicinal product has been demonstrated”).

17 UNCTAD (2015), p. 3.

18 Medicines can only be marketed after they have been approved under either national or Community marketing authorisation procedures, which confirm their safety and efficacy. The procedure is regulated by Regulation (EC) No. 726/2004 “Laying down Community procedures for the authorisation and supervision of medicinal products for human and veterinary use and establishing a European Medicines Agency” and Directive 2004/27/EC amending Directive 2001/83/EC on the Community Code relating to medicinal products for human use. Generics must also obtain a marketing authorisation; however, “a generic applicant for marketing authorisation is exempted from the requirement to prove safety and efficacy through pre-clinical tests and clinical trials, and the competent authority relies on the proof of safety and efficacy provided by the reference [originator] product” (European Commission (2009a, b, c), pp. 7–8). This means that they only need to provide evidence that their generic version is “essentially similar” to the original product.

19 *See e.g.* Roin (2009), p. 545 (“More than firms in any other industry, pharmaceutical companies rely on the patent system to secure a return on their R&D investments, particularly the large investments they make in clinical trials”); Ho (2015), p. 310 (“It is widely recognized that the pharmaceutical industry is unique among most industries in that patents are considered essential”); Commission on Intellectual Property Rights (2002), p. 29 (“The pharmaceutical industry in developed countries is more strongly dependent on the patent system than most other industrial sectors to recoup its past R&D costs, to generate profits, and to fund R&D for future products”).

20 European Commission (2009a, b, c), para. 253, citing EFPIA (“Given the clear disparity between the high cost and risk of innovation in the pharmaceutical sector and the low cost and risk of imitation, it is self-evident that exclusivity and thus protection from imitation is needed if there is to be innovation”).

21 *See* text accompanying *supra* note 18.

22 UNCTAD (2015), p. 14; OFT Decision in Case No. CE/8931/08 (12 April 2011) (*Gaviscon*) (“In the EU generic medicines typically come onto the market at prices that are about 25 per cent lower than the price of the originator product immediately prior to the loss of exclusivity. Generic entry also has the effect of decreasing the price of the originator product. In markets where generic entry occurs, average prices drop by almost 20 per cent one year after the loss of exclusivity and about 25 per cent after two years. In some cases the decrease can be as much as 80–90 per cent”); *see also* FTC (2011), p. ii.

23 European Commission (2009a, b, c), p. 6 (“Blockbuster medicine” is defined as being one that achieves annual revenues of over US$1 billion at global level).

24 Cheng (2008).

25 European Commission (2009a, b, c), para. 138 (“during the lead identification/optimisation process, a company will begin to consider filing a patent application … these applications will be concerned with the active molecules themselves … the resulting patents are often referred to as ‘primary patents’ because they relate to the first patents for the active molecules. Later during the development phase and, … not uncommonly after the product launch, further patent applications will be made for other aspects of these active molecules … [s]uch patents … are often referred to as ‘secondary patents’”).

26 European Commission (2009a, b, c), para. 486.

27 *Ibid*.; Ho (2015), p. 313.

28 European Commission (2009a, b, c), para. 487.

29 *See* text accompanying *supra* note 25.

30 Case CE-9531/11 – *Paroxetine* (2016), paras. 3.70–3.71.

31 *Ibid*., para. 3.71.

32 *Ibid*.

33 FDA (2007), p. 1 (“A drug substance may exist in many polymorphic forms”); Srivastava (2004), p. 339 (“Generic drugs may be pharmaceutically and therapeutically equivalent to a brand-name drug without infringing the innovator patents that the drug references”).

34 European Commission (2009a, b, c), para. 467 (“the term ‘patent strategies’ should be understood to encompass all strategies of a company concerning the use of the patent system to the benefit of the company in relation to generic competition. The term includes strategies on the timing and scope of filing as well as the manners in which patents are applied for”).

35 European Commission (2009a, b, c), para. 128.

36 *Gaviscon*, *supra* note 22, para. 2.77.

37 Burdon and Sloper (2003), p. 227.

38 Hemphill and Sampat (2011) (their study “provide[s] the first comprehensive evidence that allegations of attempted evergreening are real, and that such efforts have grown over time”); Kapczynski et al. (2012) (“Secondary patents provide substantial additional patent life in the pharmaceutical industry…. Evidence that they are also more common for best-selling drugs is consistent with accounts of active ‘life cycle management’ or ‘evergreening’ of patent portfolios in the industry”).

39 Ho (2015), p. 314; Myers (2008), p. 774.

40 Bansal et al. (2009), p. 299.

41 European Commission (2009a, b, c), p. 8 (“INN is the International Non-proprietary Name for pharmaceutical substances”).

42 European Commission (2009a, b, c), para. 488.

43 *Ibid*., para. 489.

44 *Ibid*., para. 492 (the EU Commission calls this practice “patent thickets” or “patent clusters”); *see*, however, Gurgula (2017) (discussing the difference between the concept of patent thickets in complex technologies and strategic accumulation of patents employed by pharmaceutical companies).

45 Ho (2015), p. 314; Kapczynski et al. (2012), p. 1.

46 European Commission (2009a, b, c), para. 1118.

47 European Commission (2009a, b, c), para. 994; FTA (2003), p. 9, citing the National Institute for Health Care Management, “Changing Patterns of Pharmaceutical Innovation” (2002), p. 7.

48 UNCTAD (2015), p. 6.

49 *Ibid*., p. 7.

50 *Ibid*.

51 *Ibid*.; Abud et al. (2015), p. 2; Ho (2015), p. 314.

52 European Commission (2019), p. 17.

53 EU Commission **(**2009a, b, c/C 45/02), para. 5.

54 OECD (2007).

55 Lianos (2016), p. 3.

56 Hettinger (1989), pp. 47–51; Machlup and Penrose (1950), p. 21.

57 Machlup and Penrose (1950), p. 21.

58 Anderman and Schmidt (2007), p. 37.

59 *See e.g.* C-274/11 and C-295/11 *Spain and Italy v Council*, EU:C:2013:240, para. 22.

60 Anderman and Schmidt (2007), p. 12.

61 *Ibid*., p. 14.

62 *Ibid*., p. 37.

63 *Ibid*., pp. 14–15 (explaining that this approach “draws some support from the work of Kenneth Arrow and others who maintain that competition may provide more incentives for innovation than monopoly”).

64 *Ibid*., p. 12.

65 Hovenkamp et al. (2016), Sect. 1.03.

66 Guidelines on the application of Article 101 Treaty on the Functioning of the EU to technology transfer agreements (2014/C 89/03), para. 7; similarly, *see* Department of Justice and the Federal Trade Commission in the US, “Antitrust Enforcement and IPRs: Promoting Innovation and Competition” (2007), p. 1 (“antitrust enforcers and the courts have come to recognize that intellectual property laws and antitrust laws share the same fundamental goals of enhancing consumer welfare and promoting innovation”).

67 Anderman and Schmidt (2007), pp. 12–13.

68 *Ibid*., p. 13.

69 *Ibid*.

70 T-321/05 *AstraZeneca v Commission* [2010] ECR II-2805; 5 CMLR 1585, para. 367.

71 *ITT Promedia v EC Commission* [1998] ECR II-2937; C-241/91 P and C-242/91 P *RTE and ITP v Commission* [1995] ECR I-743; [1995] 4 CMLR 718 (*Magill*).

72 T-201/04 *Microsoft Corp v Commission* [2007] ECR II-3601; [2006] 4 CMLR 311.

73 Case AT.40220 *Qualcomm (Exclusivity Payments)*, Commission decision of 24 January 2018.

74 The Long-Term Evolution standard.

75 European Commission (2009a, b, c), p. 9; Summary of Commission Decision of 24 January 2018 relating to a proceeding under Article 102 of the Treaty on the Functioning of the European Union and Article 54 of the EEA Agreement (Case AT.40220 – *Qualcomm (Exclusivity Payments)*).

76 Press Release, “Antitrust: Commission fines Qualcomm €997 million for abuse of dominant market position” (24 January 2018) <https://ec.europa.eu/commission/presscorner/detail/en/IP_18_421> ac-cessed 20 March 2020.

77 Case AT.40099 *Google Android*, Commission decision of 18 July 2018 <https://ec.europa.eu/competition/elojade/isef/case_details.cfm?proc_code=1_40099> accessed 20 March 2020.

78 Press Release, “Antitrust: Commission fines Google €1.49 billion for abusive practices in online advertising” (20 March 2019) <http://europa.eu/rapid/press-release_IP-19-1770_en.htm> accessed 20 March 2020. *See also* the discussion of this case in European Commission (2019), p. 9.

79 Summary of Commission Decision of 4 May 2017 relating to a proceeding under Article 102 of the Treaty on the Functioning of the European Union and Article 54 of the EEA Agreement (Case AT.40153 – *E-Book MFNS and related matters*), para. 8. The European Commission adopted a decision that rendered legally binding the commitments offered by Amazon, which addressed the Commission’s preliminary competition concerns relating to a number of clauses in Amazon’s distribution agreements with e-book publishers in Europe. *See* Press Release, “Antitrust: Commission accepts commitments from Amazon on e-books” (4 May 2017) <https://ec.europa.eu/commission/presscorner/detail/en/IP_17> accessed 20 March 2020.

80 Summary of Commission Decision of 4 May 2017 in Case AT.40153, *supra* note 79, para. 12 (for example, it considered that “the business model parity and the respective notification provisions are capable of (i) reducing e-book suppliers’ incentives to support and invest in alternative new and innovative business models; (ii) reducing Amazon’s competitors’ ability and incentives to develop and differentiate their offerings through such business models; (iii) deterring entry and/or expansion by e-book retailers, thus weakening competition at the e-book distribution level and strengthening Amazon’s dominant position”).

81 *See e.g. AstraZeneca*, *supra* note 70, para. 313.

82 European Commission (2009a, b, c), para. 253, citing EFPIA (“Given the clear disparity between the high cost and risk of innovation in the pharmaceutical sector and the low cost and risk of imitation, it is self-evident that exclusivity and thus protection from imitation is needed if there is to be innovation”).

83 Schumpeter (1976).

84 Lowe (2008), p. 3.

85 European Commission (2009a, b, c/C 45/02), para. 30.

86 *See* Gurgula (2019).

87 Case AT.39612 – *Perindopril (Servier)* [2014] (*Perindopril*), para. 122.

88 *Ibid*., para. 123.

89 Ullrich (2013), pp. 265–266.

90 COMP/A. 37.507/F3 – *AstraZeneca* [2005], para. 274.

91 UNCTAD (2015), p. 7; Abud et al. (2015), p. 2; Ho (2015), p. 314.

92 European Commission (2009a, b, c), para. 523; Correa (2015); Kesselheim (2007); Eisenberg (2008); Hemphill and Sampat (2011).

93 Ullrich (2013), p. 242 (internal citation omitted).

94 *AstraZeneca*, *supra* note 70, para. 367.

95 *Ibid*.

96 European Commission (2009a, b, c), para. 525.

97 *Ibid*.

98 *Perindopril*, *supra* note 87, para. 2815.

99 European Commission (2009a, b, c), para. 530.

100 *Ibid*.

101 *Ibid*., para. 528.

102 Maggiolino (2011), p. 92.

103 *Ibid*., p. 98.

104 Maggiolino (2011), p. 101.

105 C-56/64 and 58/64 *Consten and Grundig v Commission* [1966] ECR 299.

106 Case T-691/14 *Teva UK and Others v Commission* (12 December 2018), para. 201.

107 *Ibid*., referring to the judgment of 6 October 1982, *Coditel and Others*, 262/81, EU:C:1982:334, para. 13.

108 *Ibid*., para. 204, referring to the judgment of 31 October 1974, *Centrafarm and de Peijper*, 15/74, EU:C:1974:114, paras. 39 and 40.

109 *See e.g. Magill*, *supra* note 71; C-418/01 *IMS Health GmbH & Co OHG v NDC Health GmbH & Co KG* [2004] ECR I-5039.

110 Kjølbye (2009), p. 180 (discussing a “plus factor”); *see also* Report of the Attorney General’s National Committee to Study the Antitrust Laws (1955), p. 337 (“violation of the Sherman Act should … require abuse of the patent grant or proof of intent to monopolize beyond the lawful patent grants”).

111 *AstraZeneca*, *supra* note 70.

112 *ITT Promedia*, *supra* note 71.

113 *Magill*, *supra* note 71; *IMS Health*, *supra* note 109.

114 *Perindopril*, *supra* note 87, para. 2766.

115 *Perindopril*, *supra* note 87, para. 2766.

116 *See e.g*. Kjølbye (2009).

117 Report of the Attorney General’s National Committee to Study the Antitrust Laws (1955), p. 337.

118 Kjølbye (2009), p. 181.

119 *Ibid*.

120 *Ibid*.

121 *Ibid*.

122 *Ibid*.

123 *Ibid*., p. 182.

124 Kjølbye (2009), p. 182; Jacob (2013), p. 206; Straus (2010), p. 198.

125 Siegel (1967), p. 76.

126 *Ibid*.

127 Kjølbye (2009), p. 182; Jacob (2013), p. 206; Straus (2010), p. 198.

128 Kjølbye (2009), p. 186.

129 *Ibid*., p. 180.

130 Anderman and Ezrachi (2011), p. 7.

131 Kjølbye (2009), p. 188; European Commission (2009a, b, c), para. 253.

132 Kjølbye (2009), pp. 180–181; Straus (2010), p. 198.

133 *AstraZeneca*, *supra* note 70, para. 362.

134 *AstraZeneca*, *supra* note 70, para. 362.

135 *Ibid*.

136 Whish and Balley (2015), p. 840; 24/67 *Parke Davis & Co v Probel* [1968] ECR 55.

137 *AstraZeneca*, *supra* note 70, para. 677.

138 Ullrich (2013), p. 258.

139 *See* Gilbert (1987), p. 209 (arguing that a firm may be able to protect and maintain its monopoly position by developing and patenting substitute technologies. Gilbert explains that the protection of monopoly power through internal research and development, may be more profitable than the acquisition of exclusive licences from potential rivals).

140 Ullrich (2013), p. 263.

141 *See United States v Aluminum Co. of America*, 148 F.2d 416 (2d Cir. 1945) (where judge Learned Hand observed that “drastic expenditures of research and development with knowledge or intent to pre-empt and dominate an industry” may raise competition law concerns).
